# Knockdown of CK2α reduces *P*-cresol-induced fibrosis in human renal proximal tubule epithelial cells via the downregulation of profilin-1

**DOI:** 10.7150/ijms.48429

**Published:** 2020-10-16

**Authors:** Yeo Min Yoon, Gyeongyun Go, Chul Won Yun, Ji Ho Lim, Sang Hun Lee

**Affiliations:** 1 Medical Science Research Institute, Soonchunhyang University Seoul Hospital, Seoul, 04401, Republic of Korea.; 2 Department of Biochemistry, Soonchunhyang University College of Medicine, Cheonan, 31151, Republic of Korea.

**Keywords:** CK2α, Profilin-1, CKD, fibrosis, stress F-actin, renal proximal tubule epithelial cell

## Abstract

Renal fibrosis is one of the main causes of chronic kidney disease. Many studies have focused on fibroblasts and myofibroblasts involved in renal fibrogenesis. Recently, several studies have reported that renal proximal tubule epithelial cells are possible initiators of renal fibrosis. However, the mechanism through which cells induce renal fibrosis is poorly understood. In this study, we found that CK2α induces fibrosis in renal proximal tubule epithelial cells (TH1) by regulating the expression of profilin-1 (Pfn1). CKD mouse model and TH1 cells treated with *P*-cresol also showed an increased level of Pfn1. The knockdown of CK2α suppressed fibrosis in TH1 cells via the downregulation of Pfn1. In particular, CK2α knockdown inhibited the expression of stress fibers and fibrosis-related proteins in *P*-cresol-treated TH1 cells. Furthermore, the knockdown of CK2α inhibited mitochondrial dysfunction and restored cellular senescence and cell cycle in *P*-cresol-treated TH1 cells. These results indicate that CK2α induces renal fibrosis through Pfn1, which makes CK2α a key target molecule in the treatment of fibrosis related to chronic kidney disease.

## Introduction

Chronic kidney disease (CKD), caused by increased risk of fibrosis and the progressive loss of kidney function, represents a global health concern due to increasing pervasiveness (8-16%) of the disease 1. The origin of persistent injury response and repair signaling underlying fibrotic tissue destruction is poorly understood. Evidence has shown that renal proximal tubule epithelial cells are possible initiators of renal fibrosis, which leads to different types of injury 2, 3. Renal proximal tubule epithelial cells, one of the major components of the kidney, are vulnerable to different types of injury including proteinuria, toxins, metabolic disorders, and senescence 2, 3. In CKD patients, the damaged kidney secretes uremic toxics such as indoxyl sulfate and *P*-cresol, and the accumulation of these toxins causes various complications. Our previous studies proved that *P*-cresol, a low molecular weight uremic toxin with high affinity to protein, reduced cellular functions by various mechanisms, including accumulated reactive oxygen species (ROS), cell senescence, abnormal mitochondria, and endoplasmic reticulum stress 4-6. Therefore, in the treatment of CKD patients, it is important to protect renal proximal tubule epithelial cells against damages caused by uremic toxins.

Actin stress fibers (F-actin) are exquisitely sensitive to the dynamically changing mechanical environment, and they are essential in the regulation of cell polarization and transmission of tensional force from focal adhesion to the cells 7. Some studies have indicated that stress fiber induces the expression of fibrosis markers, such as α-smooth muscle actin and vimentin, and differentiation into fibroblasts in idiopathic pulmonary fibrosis and end-stage heart failure caused by fibrosis 8, 9. Profilin-1 (Pfn1), a well-known actin binding protein, is an important key facilitator for the regulation of actin dynamics 10. Pfn1 regulates cellular homeostasis and maintains cytoskeletal structure. However, previous clinical studies verified that Pfn1 is highly expressed in endothelial cells and in the serum of CKD patients with decreased eGFR 11. These results indicate that, in CKD patients, the overexpression of Pfn1 disrupts the homeostasis of actin dynamics and increase F-actin in renal proximal tubule epithelial cells. Therefore, we hypothesized that the inhibition of the key upstream regulator of Pfn1 could suppress fibrosis in renal proximal tubule epithelial cells.

Casein kinase 2 (CK2), a serine/threonine kinase, is a heterotetrameric complex consisting of two catalytic subunits (CK2α/α') and two regulatory subunits (CK2β) 12, 13. These CK2 subunits act as constitutive enzymes to phosphorylate several proteins associated with various cellular processes, including proliferation, differentiation, apoptosis, and DNA repair 12, 13. CK2 regulates actin-myosin II-base contractility at Ser^1943^ and cytoskeleton through co-localization with F-actin 14, 15. In idiopathic pulmonary fibrosis, abnormally high activity of CK2 increased fibrosis-associated proteins and DNA repair activity 16. Elevated protein level and kinase activity of CK2α enhance fibrosis and increase the expression of inflammatory proteins such as NF-κB 17, 18. In this study, we found that the knockdown of CK2α in human renal proximal tubular epithelial cells (TH1) restored stress fibers and decreased the expression of fibrosis markers, such as α-SMA and fibronectin, via the downregulation of Pfn1. Furthermore, we found that the knockdown of CK2α restored cellular functions, such as mitochondria metabolism, senescence, and cell cycle. These results suggest that CK2a is an important target in the treatment of CKD through the inhibition of fibrosis.

## Materials and Methods

### Human renal proximal tubule epithelial (THI) cell culture

Human renal proximal tubule epithelial cell line (TH1) was purchased from Kerafast (Boston, MA, USA). Cells were cultured in Minimum Essential Medium (Thermo Fisher Scientific, Waltham, MA, USA) supplemented with 10 % (v/v) fetal bovine serum (Thermo Fisher Scientific) and 100 U/mL penicillin/streptomycin (Thermo Fisher Scientific). Cells were grown in a humidified incubator at 37 °C and 5 % CO_2_. TH1 cells at passage 6-7 were used for the experiments.

### Detection of Pfn1 in serum and cell lysate

The concentrations of Pfn1 in mouse serum or cell lysates were determined by using an ELISA kit (Cusabio, Hebei, China). Serum sample (100 μL) from each group was analyzed to determine Pfn1 expression. The wells were quantified by measuring absorbance at 450 nm using a microplate reader (BMG Labtech, Ortenberg, Germany).

### Western Blotting

The whole lysates (30-50) μg of sample protein were subjected to sodium dodecyl sulfate-polyacrylamide gel electrophoresis in 8-12 % gel, and transferred to polyvinylidene fluoride membranes. After blocking with 5 % skim milk in TBST (10 mM Tris-HCl (pH 7.6), 150 mM NaCl, 0.05 % Tween-20), the membranes were immunoblotted with the primary antibodies against CK2α (1:1000), Pfn1 (1:1000), Myosin-X (1:1000), Myosin IIA (1:1000) (NOVUS, Littleton, CT, USA), fibronectin (1:1000), α-SMA (1:1000) (Thermo Fisher Scientific), p-Myosin IIA (1:1000), p-P53 (1:1000), P16 (1:1000) (Cell signaling, Danvers, MA, USA), P21 (1:1000), SMP30 (1:1000), cyclin D1 (1:1000), CDK4 (1:1000), cyclin E (1:1000), CDK2 (1:1000), and β-actin (1:1000) (Santa Cruz Biotechnology, Dallas, TX, USA). The membranes were then washed, and the primary antibodies were detected by means of goat anti-rabbit IgG (1:10,000) or goat anti-mouse IgG antibodies (1:10,000) (Santa Cruz Biotechnology). The bands were detected by enhanced chemiluminescence (Thermo Fisher Scientific). Band densitometric quantifications were determined using ImageJ software 1.48v.

### Measurement of oxygen consumption rate (OCR) and equated to the glycolytic rate (ECAR)

OCR and ECAR were measured in TH1 cells using the XF96 Extracellular Flux analyzer (Seahorse Bioscience, MA, USA). Briefly, TH1 cells were seeded at 45,000 cells per well in XF96 cell culture multi-well plates in DMEM medium and incubated for 24 h in incubator at 37 °C and 5 % CO_2_. Next, XF96 cartridges were incubated overnight in XF calibrant at 37 °C in a non-CO_2_ incubator. After the growth medium of TH1 cells were exchanged with XF medium, the plates were incubated at 37 °C in a non-CO_2_ incubator for 1 h. Inhibitors were diluted to appropriate concentrations in XF medium and loaded into corresponding microwells in the XF96 cartridge plate. Following the equilibration of sensor cartridges, XF96 cell culture plate was loaded into the XF96 Extracellular Flux analyzer at 37 °C. OCR and ECAR were measured after cycles of mixing and data acquisition (basal) or inhibitor injection, mixing, and data acquisition.

### Senescence-associated β-galactosidase assay

Senescence-associated β-galactosidase activity was analyzed using a Senescence β-Galactosidase Staining Kit (Cell Signaling Technology).

### Cell cycle analysis

Untreated TH1 cells, *P*-cresol-treated TH1 cells, and *P-*cresol-pretreated TH1 cells after treatment with si-CK2α or si-Scr were trypsinized and fixed with 70 % ethanol at -20 °C for 2 h. Next, the groups were incubated with RNase and the DNA-intercalating dye propidium iodide (PI; Sysmex) at 4 °C for 1 h. PI-stained groups were characterized by flow cytometry (Sysmex). Events were recorded for at least 10,000 cells per sample. The sample data were analyzed using the FCS express 5 software (DeNovo Software). Independent experiments were carried out thrice.

### Phalloidin staining

Phalloidin staining (Santa Cruz Biotechnology) was performed with 4′,6-diaminido-2-phenylindol (DAPI; Sigma Aldrich, MO, USA), and the immunostained samples were examined by confocal microscopy (Olympus, Tokyo, Japan).

### Ethics statement

All animal care procedures and experiments were approved by the Institutional Animal Care and Use Committee of Soonchunhyang University Seoul Hospital (IACUC2013-5) and were performed in accordance with the National Research Council (NRC) Guidelines for the Care and Use of Laboratory Animals. The experiments were performed on 8-week-male BALB/c nude mice (Biogenomics, Seoul, Korea) maintained on a 12-h light/dark cycle at 25 °C in accordance with the regulations of Soonchunhyang University Seoul Hospital**.**

### CKD mouse model

Eight-week-old male BALB/c nude mice were fed an adenine-containing diet (0.75 % adenine in diet) for 1-2 weeks. Mouse body weight was measured every week. If the body weight of the mouse was rapidly decreased to 15-16 g, the adenine feeding was stopped. After 2-3 days, if there were no further changes in body weight, the mouse was sacrificed. After euthanasia, blood was stored at -80 °C for the measurement of blood urea nitrogen (BUN) and creatinine.

### Hemotoxylin and Eosin staining (H&E) and picrosirius red staining

At 2 week after feeding the mice the adenine-containing diet, the renal tissues were removed and fixed with 4 % paraformaldehyde (Sigma Aldrich), and each tissue sample was embedded in paraffin. For histological analysis, the samples were stained with H&E to determine fibrosis and examine histopathological features. The diameter of glomerulus was blindly measured from multiple slides per sample and average diameter of glomerulus determined by using ImageJ software (NIH; version 1.43).

### Statistical analysis

Results are expressed as the mean ± standard error of the mean (SEM) and evaluated by two-tailed Student's t-test or one- or two- way analysis of variance (ANOVA) to compute the significance between the groups. Comparisons of three or more groups were made by using Dunnett's or Tukey's post-hoc test. Data were considered significantly different at *p* < 0.05.

## Results

### Profilin-1 is increased in the CKD mouse model

**Figure 1:**
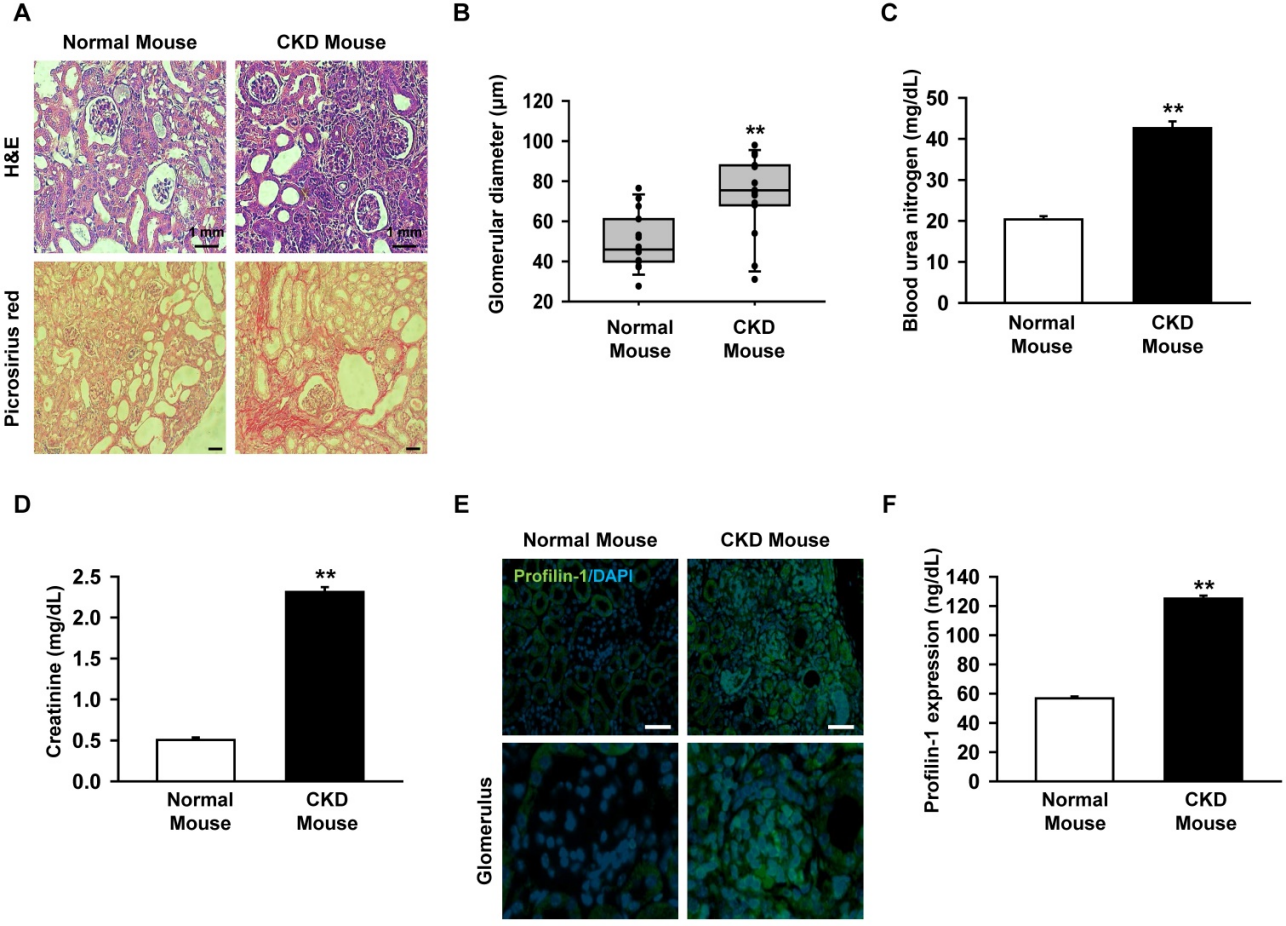
Expression of profilin-1 (Pfn1) is increased in CKD mouse model. (A) CKD mouse model that was fed with 0.75 % adenine for 2 weeks, and we examined mouse renal glomerulus through H&E and picrosirius red staining. Scale bar = 1 mm. (B) The diameter of glomerulus in the kidney of CKD mouse. Values represent the mean ± SEM. **p < 0.01 vs. normal mouse (n = 15). (C-D) Measurement of blood urea nitrogen (BUN) and creatinine in mouse serum with enzyme-linked immunosorbent assay (ELISA). Values represent the mean ± SEM. **p < 0.01 vs. normal mouse (n = 3). (E) Immunofluorescence staining of the kidney of CKD mouse with Pfn1. (F) Detection of the expression of Pfn1 in CKD mouse serum using ELISA. Values represent the mean ± SEM (n = 3). **p < 0.01 vs. normal mouse (n = 3).

Previous clinical studies reported that the expression of Pfn1 is highly increased in the serum of CKD patients with a low recovery rate 11. We assumed that renal fibrosis could be inhibited by downregulating the upstream regulator of Pfn1. To make the CKD mouse model, mice were fed with 0.75% adenine for 1-2 weeks. We confirmed that the glomerulus size increased and fibrosis occurred in the CKD mice using H&E and picrosirius red staining (Figure 1A-B). In addition, BUN and creatinine levels were increased in mouse serum as previously reported (Figure 1C, and 1D) 19. To confirm that Pfn1 expression is increased in the CKD mouse model, the Pfn1 expression levels in kidney and the serum were analyzed. Immunofluorescence staining of kidney tissue with Pfn1 showed that higher expression of Pfn1 in the glomerulus of the CKD mice compared to that of normal mice (Figure 1E). Furthermore, the CKD mice showed higher expression of Pfn1 in the serum samples than normal mice (Figure 1F). These data indicate that the epithelial cells in the glomerulus may have high expression of Pfn1 due to enhanced fibrosis. Thus, we focused on the increased expression of Pfn1 in renal proximal tubule epithelial cells (TH1) which are susceptible to fibrosis in CKD.

### Profilin-1 is increased in P-cresol-treated TH1 cells via a CK2α upstream regulator

**Figure 2:**
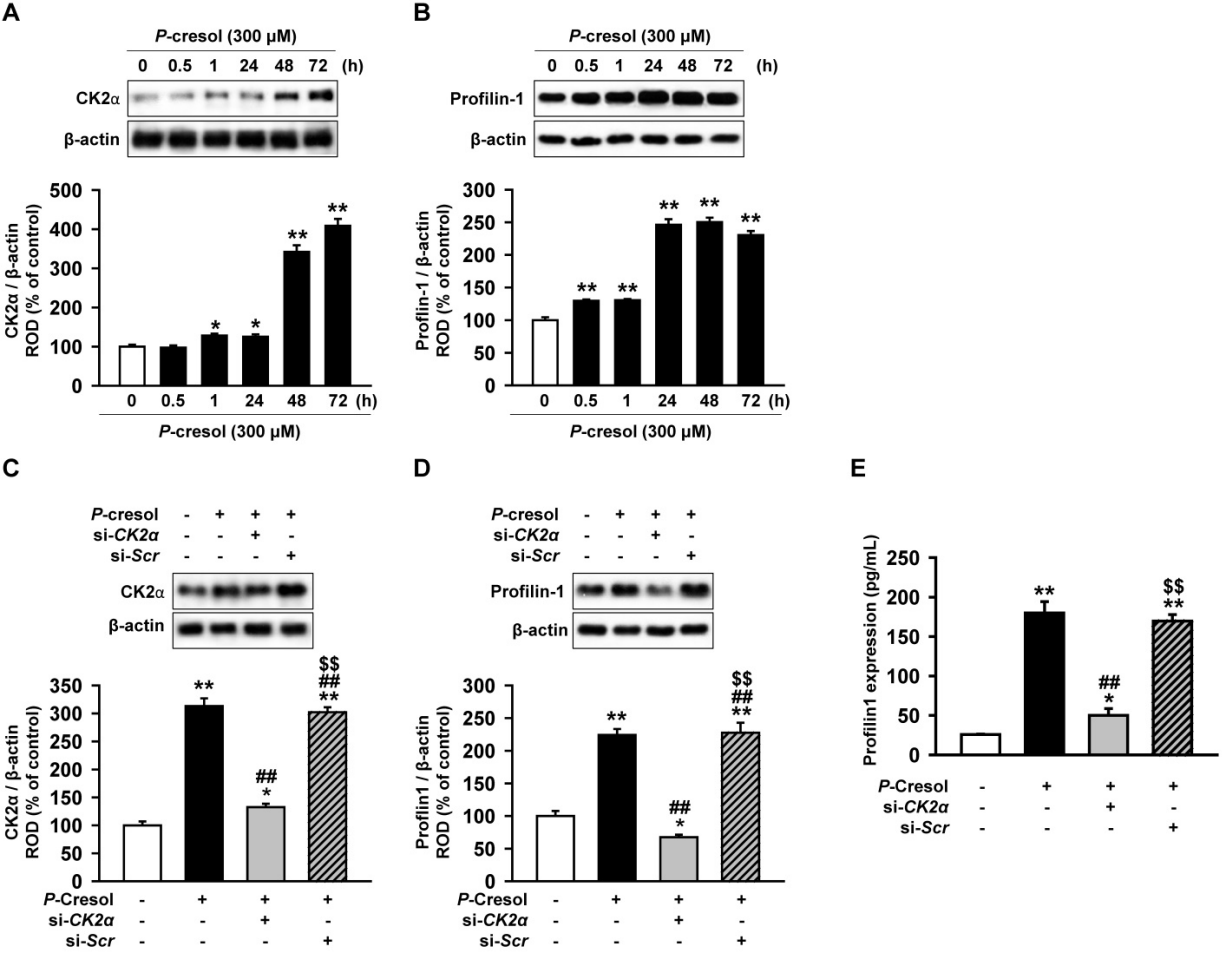
Knockdown of CK2α in TH1 cells decreases Pfn1 expression in *P*-cresol exposure condition. (A-B) Western blot analysis of the expression of CK2α and Pfn1 in TH1 cells exposed to *P*-cresol (300 µM) for 0, 0.5, 1, 24, 48, and 72 h. The expression levels relative of that of β-actin were determined by densitometry. Values represent the mean ± SEM. *p < 0.05, and **p < 0.01 vs. untreated TH1 (n = 3). (C-D) Expression of CK2α and Pfn1 were analyzed in pretreated TH1 cells with si-*CK2α* or si-*Scr* in *P*-cresol exposure condition (300 µM, 72 h). The expression levels relative of β-actin were determined by densitometry. Values represent the mean ± SEM. *p < 0.05, and **p < 0.01 vs. untreated TH1, ^##^*p <* 0.01 vs. *P*-cresol exposure condition, ^$$^*p <* 0.01 vs. knockdown of CK2α in TH1 cells in *P*-cresol exposure condition (n = 3). (E) Expression of Pfn1 was measured in pretreated TH1 cells with si-*CK2α* or si-*Scr* in *P*-cresol exposure condition (300 µM, 72 h). Values represent the mean ± SEM (n = 3). *p < 0.05, and **p < 0.01 vs. untreated TH1, ^##^*p <* 0.01 vs. *P*-cresol exposure condition, ^$$^*p <* 0.01 vs. knockdown of CK2α in TH1 cells in *P*-cresol exposure condition (n = 3).

CK2α is well known to regulate actin stress fibers (F-actin) and induces fibrosis 18, 20. However, it is not known whether CK2α regulates Pfn1. In order to confirm that the expression of CK2α in TH1 cells is changed in CKD environment, expression of CK2α was measured in TH1 cells treated with *P*-cresol, a uremic toxin associated with CKD. TH1 cells showed increased expression of CK2α upon exposure to *P*-cresol for 30 min and 1, 24, 48, and 72 h (Figure 2A). The expression of Pfn1, the regulatory protein for F-actin cytoskeleton reorganization, was also increased by *P*-cresol exposure in a time-dependent manner (30 min, and 1, 24, 48, 72 h), as observed from the western blot (Figure 2B). To confirm that CK2α is the key regulator of Pfn1, we checked the expression of Pfn1 after CK2α siRNA treatment. The expression of Pfn1 was decreased by the knockdown of CK2α in TH1 cells exposed to *P*-cresol (Figure 2C, 2D, and 2E). Thus, we proved that the knockdown of CK2α in TH1 cells reduced the expression of Pfn1 which possibly restored cellular functions and fibrosis in TH1 cells exposed to *P*-cresol.

### Knockdown of CK2α in TH1 cells restores mitochondria metabolism in the P-cresol exposure condition

**Figure 3:**
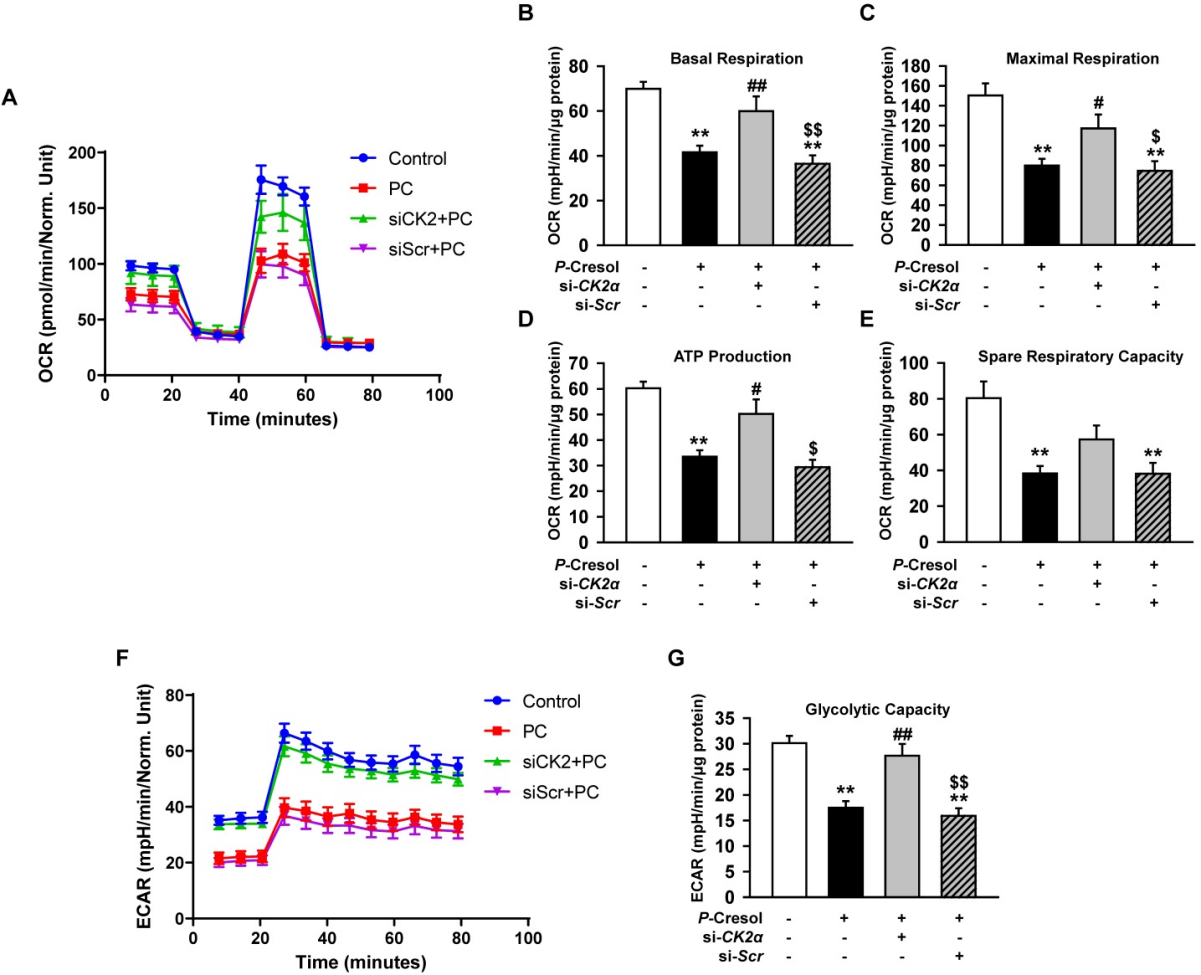
Knockdown of CK2α in TH1 cells restores mitochondrial function in *P*-cresol exposure condition. (A-E) Oxygen consumption rate (OCR) of TH1 cells treated with CK2α or scramble siRNA in *P*-cresol exposure condition (300 µM, 72 h) (n = 4). (F-G) Equated to the glycolytic rate (ECAR) of TH1 cells-treated with CK2α or scramble siRNA in *P*-cresol exposure condition (300 µM, 72 h). Values represent the mean ± SEM. **p < 0.01 vs. untreated TH1, ^#^*p <* 0.05, and ^##^*p <* 0.01 vs. *P*-cresol exposure condition, ^$^*p <* 0.05, and^ $$^*p <* 0.01 vs. knockdown of CK2α in TH1 cells in *P*-cresol exposure condition (n = 4).

Although the knockdown of CK2α in TH1 cells decreased the expression of Pfn1, other studies reported that CK2α also regulates cellular functions, such as mitochondrial function, senescence, and cell cycle 21, 22. To confirm the effect of CK2α on mitochondria metabolism, we measured mitochondrial respiration by using a seahorse analyzer. We showed that CK2α knockdown restored metabolism signaling by the increased OCR in si-CK2α-pretreated group compared with that in the *P*-cresol-treated group (Figure 3A-E). The knockdown of CK2α also restored glycolytic capacity in the *P*-cresol exposure condition (Figure 3F, and 3G). These results indicate that the knockdown of CK2α in TH1 cells restored ATP synthesis through mitochondrial respiration and glycolytic capacity in the *P*-cresol exposure condition without adverse effects.

### Inhibition of CK2a expression protect TH-1 cells from p-cresol-induced cellular senescence and changes in cell cycle

**Figure 4:**
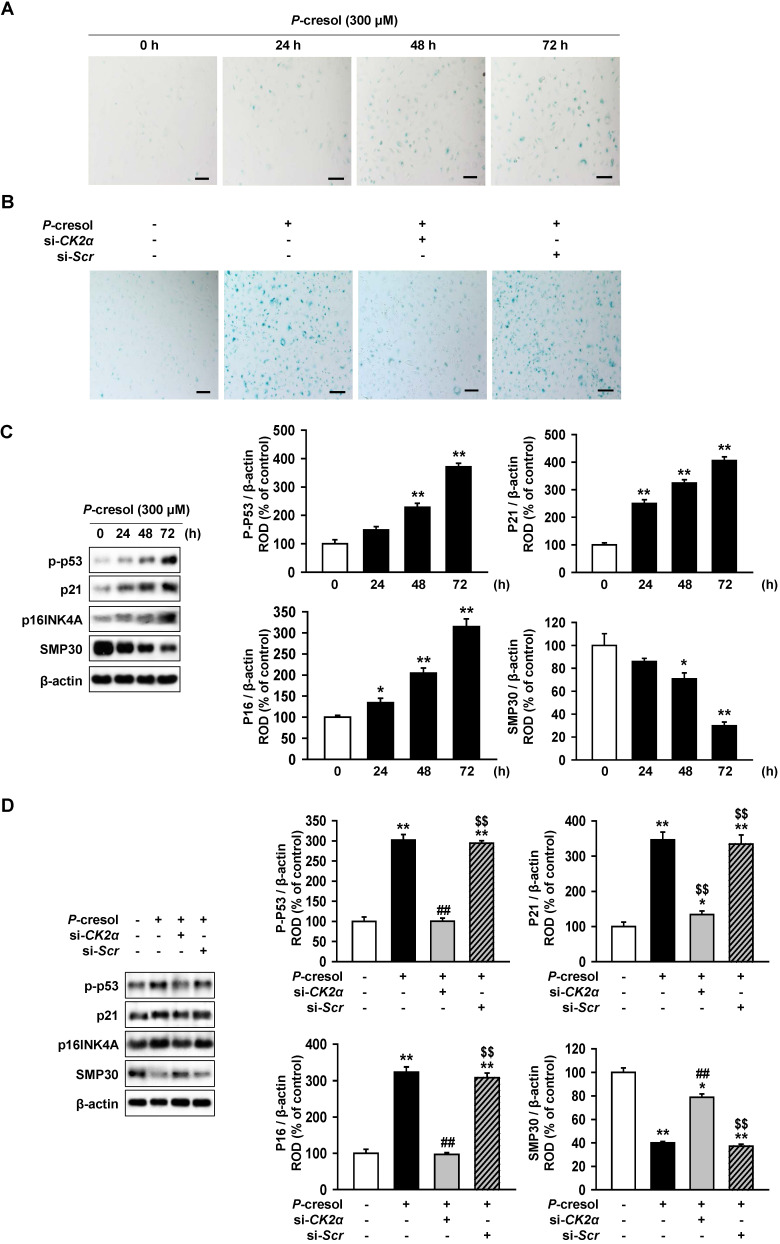
Knockdown of CK2α restore the *P*-cresol-induced cellular senescence in TH-1 cells. (A-B) We measured the senescence of β-galactosidase (β-gal)-stained TH1 cells at various time (0, 24, 48, and 72 h). (B) In *P*-cresol exposure condition (300 µM, 72 h), CK2α knockdown was determined in β-galactosidase (β-gal)-stained cells. (C) Western blotting was used to analyze the expression and activation of senescence-associated proteins, P-p53, p16 (INK4A), SMP30, and p21 in TH1 cells exposed to *p*-cresol (300 μM) for 0, 24, 48, and 72 h. The expression levels were determined by densitometry, relative to the expression of β-actin. Values represent the mean ± SEM. **p < 0.01 *vs.* untreated TH1 (n = 3). (D) Upon exposure to *p*-cresol (300 µM, 72 h), the expression and activation of senescence-associated protein, P-p53, p16 (INK4A), SMP30, and p21 were detected in CK2α knockdown TH1 cells (n = 3).

**Figure 5:**
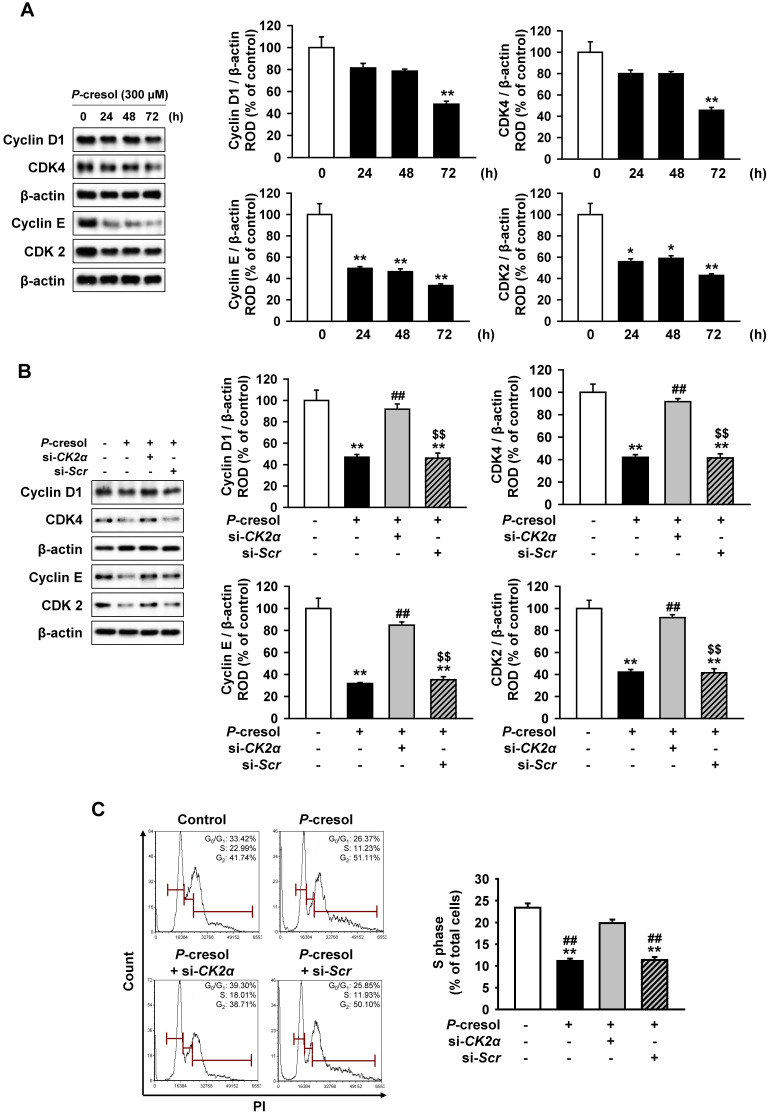
CK2α knockdown restored *P*-cresol-induced expression pattern of cell cycle-associated proteins and cell cycle change in TH-1 cells. (A) Western blotting was used to analyze the expression of cell cycle-associated proteins, Cyclin D1, Cyclin-dependent kinase 4 (CDK4), Cyclin E, and Cyclin-dependent kinase 4 (CDK2) in TH1 cells exposed to *P*-cresol (300 µM) for 0, 24, 48, and 72 h (n = 3). (B) In the *P*-cresol exposure condition (300 µM, 72 h), CK2α knockdown in TH1 cells detected the expression of cell cycle-associated proteins, Cyclin D1, CDK4, Cyclin E, and CDK2 (n = 3). (C) Cell cycle analysis in TH1 cells. Cell proliferation is quantified as the percentage of S phase in each group. Values represent the mean ± SEM. *p < 0.05, **p < 0.01 vs. untreated TH1, ^##^*p <* 0.01 vs. *P*-cresol exposure condition, ^$$^*p <* 0.01 vs. knockdown of CK2α in TH1 cells in *P*-cresol exposure condition (n = 3).

Next, we confirmed the effect of CK2α knockdown on cellular senescence and cell cycle in *P*-cresol-treated TH1 cells. *P*-cresol increased β-galactosidase (β-gal)-positive cells in time- (0, 24, 48, and 72 h) dependent manners (Figure 4A). However, CK2α knockdown restored cellular senescence in TH1 cells (Figure 4B). CK2α knockdown in TH1 cells also decreased senescence markers (p-p53, p21, and p16) and increased anti-senescence marker (SMP30), while *P*-cresol increased senescence markers in TH1 cells in a time-dependent manner (Figure 4C, and 4D). To investigate the effect of CK2α knockdown on cell cycle in *P*-cresol-treated TH1 cells, cell cycle-associated proteins (Cyclin D1, CDK4, Cyclin E, and CDK2) were analyzed by western blot. *P*-cresol treatment reduced the expression of cyclin D1, CDK4, Cyclin E, and CDK2 in time- (0, 24, 48, and 72 h) dependent manners (Figure 5A). Under *P*-cresol condition, the expression of the cycle-associated proteins was restored with CK2α knockdown, compared to control (Figure 5B). Cell cycle analysis showed that *P*-cresol induced changes in cell cycle and reduced the percentage of S phase compared to the control. CK2α knockdown protected TH1 cells from *P*-cresol mediated cell cycle change and increased the percentage of S phase (Figure 5C). These findings indicate that knockdown of CK2α protects TH-1 cells from p-cresol-induced cellular senescence and changes in cell cycle.

### Knockdown of CK2α reduces stress actin fibers in human renal proximal tubule epithelial cells through the downregulation of Pfn1

**Figure 6:**
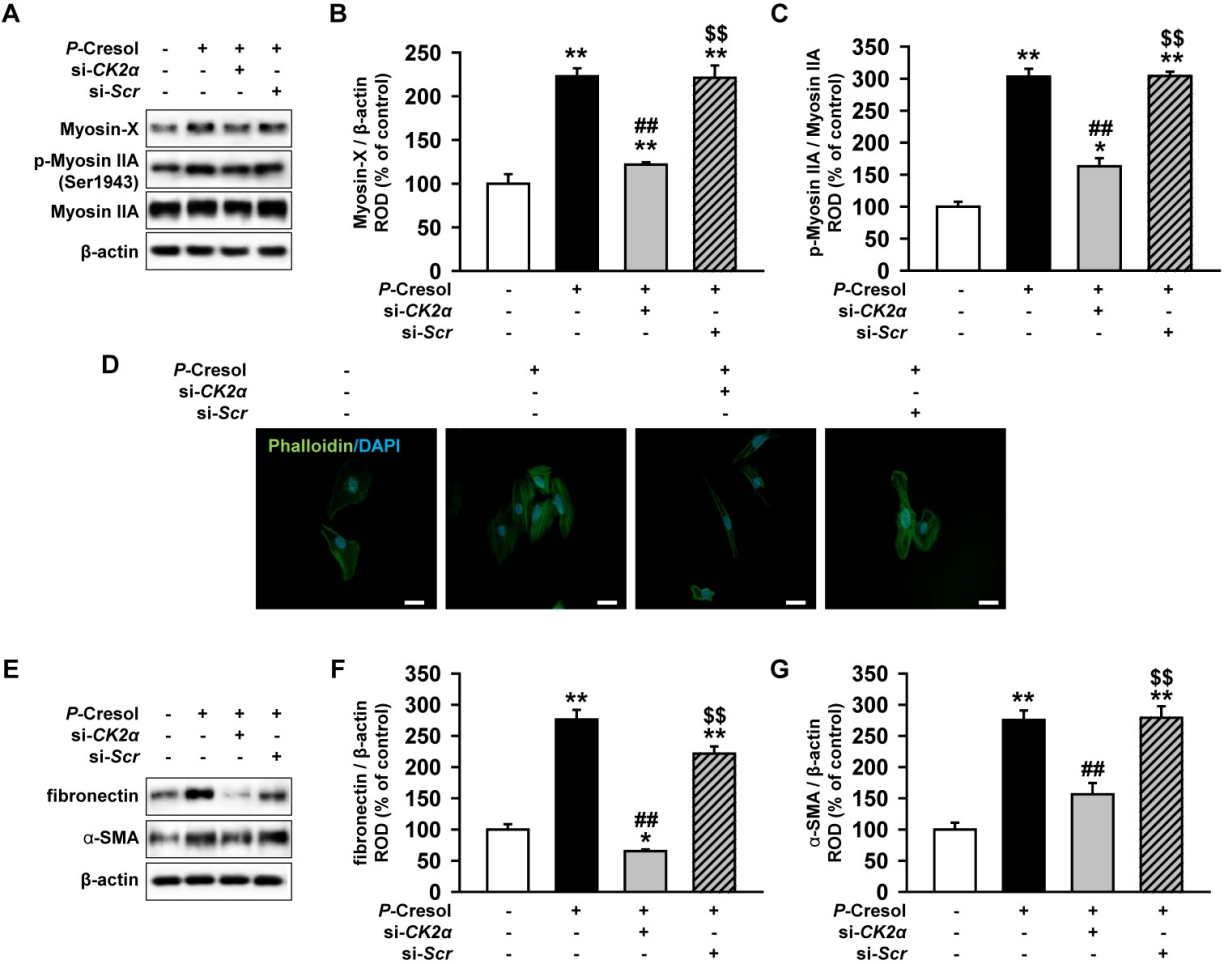
Knockdown of CK2α in TH1 cells reduces fibrosis via decreased expression of Pfn1. (A) Expression of F-actin cytoskeleton-related proteins (Myosin X and p-Myosin IIA) were measured in pretreated TH1 cells with si-*CK2α* or si-*Scr* in *P*-cresol exposure condition (300 µM, 72 h). (B-C) The expression levels were determined by densitometry, relative of Myosin IIA or β-actin, respectively. Values represent the mean ± SEM. *p < 0.05, and **p < 0.01 vs. untreated TH1, ^##^*p <* 0.01 vs. *P*-cresol exposure condition, ^$$^*p <* 0.01 vs. knockdown of CK2α in TH1 cells in *P*-cresol exposure condition (n = 3). (D) Phalloidin staining results showed F-actin expression in TH1 cells pretreated with si-*CK2α* or si-*Scr* in *P*-cresol exposure condition (300 µM, 72 h). (E) Expression of fibrosis-associated proteins (fibronectin and α-SMA) was analyzed by Western blot in TH1 cells pretreated with si-*CK2α* or si-*Scr* in *P*-cresol exposure condition (300 µM, 72 h). (F-G) The expression levels were determined by densitometry relative of β-actin. Values represent the mean ± SEM. **p < 0.01 vs. untreated TH1, ^##^*p <* 0.01 vs. *P*-cresol exposure condition, ^$$^*p <* 0.01 vs. knockdown of CK2α in TH1 cells in *P*-cresol exposure condition (n = 3).

To confirm that CK2α knockdown reduces fibrosis, we measured the expression of stress actin fibers that are composed of Myosin X and p-Myosin IIA. While TH1 cells showed the activation and increased expression of stress fiber proteins in the *P*-cresol exposure condition, the knockdown of CK2α in TH1 cells decreased stress fiber proteins (Figure 6A, 6B, and 6C). Phalloidin staining showed that CK2α knockdown also restored stress actin fibers in TH1 cells (Figure 6D). Next, using western blotting, we confirmed that the knockdown of CK2α in TH1 cells decreased the expression of fibrosis markers (fibronectin and α-SMA) (Figure 6E, 6F, and 6G). These results showed that CK2α knockdown in TH1 cells reduced fibrosis with the reorganization of the F-actin cytoskeleton by decreasing the expression of Pfn1.

## Discussion

Fibrosis is a progressive renal disorder and a shared pathological characteristic of many fatal renal diseases, such as CKD and AKI. To date, there is no effective cure for these fibrotic diseases as there is an incomplete understanding of their pathogenesis. In the progression of CKD, stress fibers in epithelial cells occur in CKD and experimental CKD models. Our CKD mouse model with an increased size of glomerulus showed high expression of Pfn1 in the serum. These results indicate that fibrosis is closely connected with stress fibers, and Pfn1 which regulates actin dynamics plays an important role in fibrogenesis. We demonstrated that CK2α knockdown restored stress fibers and inhibited fibrosis through the downregulation of Pfn1. In addition, we showed that the knockdown of CK2α restored mitochondrial function in human proximal tubular cells (TH1) in *P*-cresol exposure condition. Therefore, we discovered that the CK2α-Pfn1 signaling pathway is a potential target against fibrosis in the treatment of CKD.

Pfn1 promotes the assembly of globular-actin monomers (G-actin) into filamentous-actin (F-actin). Previous studies showed that myosin-actin interactions play a role in the increased intracellular tension of fibroblast-to-myofibroblast transition. Therefore, myosin is diffusely activated in stress fibers, extending to focal adhesions, immobilizing the fibroblast, and promoting myofibroblast differentiation in fibrotic lung progression 23-25. A clinical research reported that Pfn1 is highly expressed in endothelial cells and in the serum of patients with decreased eGFR 11. Our study also observed increased Pfn1 expression in TH1 cells treated with *P*-cresol. Thus, when the upstream regulator of Pfn1 was inhibited, fibrosis was reduced in proximal tubule epithelial cells. CK2 is composed of two catalytic subunits (CK2α and CK2α'), and both exhibit very similar enzymatic characteristics 12, 13. However, there is evidence of functional distinctions between CK2α and CK2α' due to the possibility that CK2α has a specific binding domain site to the protein phosphatase 2A 12, 13. Other studies showed that CK2α regulates cytoskeletal reorganization through the activation of Myosin IIA-associated pro-fibrotic fibroblasts 25, 26. In diabetic renal fibrosis mouse model, CK2α expression was increased in the kidney and treatment of TBB (a selective inhibitor of CK2) or CK2α RNAi adenovirus infection to the mice reduced renal fibrosis by suppressing IκB degradation and NF-κB nuclear accumulation 18.Our study proved that TH1 cells treated with *P*-cresol showed increased expression of CK2α, and the knockdown of CK2α decreased the expression of Pfn1. Thus, our study demonstrated that CK2α increases the expression of stress fiber-associated proteins, such as Myosin IIA and Myosin X, by regulating the expression of Pfn1.

It is known that mitochondrial damage in renal proximal tubule epithelial cells may induce renal fibrosis by inducing ROS formation, NLRP3 inflammasome activation, and expression of pro-inflammatory cytokines, IL-1β, and IL-18 3. A recent study demonstrated that the inhibition of CK2α reduced the expression of mitochondrial pro-apoptotic proteins and rescued the overproduction of ROS 27. In this study, we showed that the knockdown of CK2α rescued TH1 cells from *P*-cresol-induced mitochondria dysfunction, accelerated senescence, and arrested cell cycle without adverse effects 6, 28, although CK2α may regulate mitochondrial metabolism via the up-regulation of insulin receptor 29. In addition, we showed that the knockdown of CK2α reduced stress fibers and restored mitochondrial function as well as ATP synthesis. Although our data did not confirm the relationship between stress fibers and mitochondria function in CKD environment, our previous study demonstrated that mitochondrial dysfunction enhanced the progression of CKD 30. Therefore, we believe that stress fibers are first increased in TH1 cells, and mitochondrial dysfunction occurs because stress fibers regulate mitochondrial dynamics 31. In future studies, we aim to study the correlation between stress fibers and mitochondrial function, as well as determine if CK2 inhibitors, such as indenoidole, inhibit renal fibrosis.
